# Demographics and outcomes of critically ill patients transferred from other hospitals to a tertiary care academic referral center in Saudi Arabia

**DOI:** 10.1186/2110-5820-3-26

**Published:** 2013-08-11

**Authors:** Asgar H Rishu, Abdulaziz S Aldawood, Samir H Haddad, Hani M Tamim, Hasan M Al-Dorzi, Ahmed Al-Jabbary, Abdullah Al-Shimemeri, Muhammad R Sohail, Yaseen M Arabi

**Affiliations:** 1 Intensive Care Department, King Abdulaziz Medical City, Riyadh, Saudi Arabia; 2 King Abdullah International Medical Research Center, Riyadh, Saudi Arabia; 3 Intensive Care Department, College of Medicine, King Saud bin Abdulaziz University for Health Sciences, King Abdulaziz Medical City, PO Box 22490, Mail code 1425, Riyadh, 11426, Saudi Arabia

**Keywords:** Emergency department, Hospital mortality, Hospital wards, Intensive care unit, Mortality, Ambulance, Trauma

## Abstract

**Background:**

The objective of this study was to examine the outcomes of critically ill patients who were transferred from other hospitals to a tertiary care center in Saudi Arabia as a quality improvement project.

**Methods:**

This was a retrospective study of adult patients admitted to the medical-surgical intensive care unit (ICU) of a tertiary care hospital. Patients were divided according to the source of referral into three groups: transfers from other hospitals, and direct admissions from emergency department (ED) and from hospital wards. Standardized mortality ratio (SMR) was calculated. Multivariate analysis was performed to determine the independent predictors of mortality.

**Results:**

Of the 7,654 patients admitted to the ICU, 611 patients (8%) were transferred from other hospitals, 2,703 (35.3%) were direct admissions from ED and 4,340 (56.7%) from hospital wards. Hospital mortality for patients transferred from other hospitals was not significantly different from those who were directly admitted from ED (35% vs. 33.1%, *p =* 0.37) but was lower than those who were directly admitted from hospital wards (35% vs. 51.2%, *p* < 0.0001). SMRs did not differ significantly across the three groups.

**Conclusions:**

Critically ill patients who were transferred from other hospitals constituted 8% of all ICU admissions. Mortality of these patients was similar to patients with direct admission from the ED and lower than that of patients with direct admission from hospital wards. However, risk-adjusted mortality was not different from the other two groups.

## Background

Transfer of patients from one hospital to another, usually a referral center, for more advanced care is a common practice to provide access to qualified staff, specialized services, and sophisticated technologies. Studies have shown that transferring critically ill patients to specialized centers may be associated with improved outcomes [1,2]. However, several studies have suggested that referral of patients may be associated with worse outcomes, including increased mortality and morbidity [3-6], low benchmark performance of the referral centers [3,4], increased resource utilization [7,8], and increased infections [9]. On the other hand, a study of patients transferred from rural hospitals to the intensive care unit (ICU) of a tertiary-care hospital showed no difference in mortality compared with those transferred from wards within the hospital [10]. Similarly, a recent meta-analysis of trauma patients showed no difference in mortality among transferred and direct admissions [11].

Some of the variability in the observed outcomes may lie in the differences in transfer practices among centers and countries. While most studies have been reported from North American and European countries [3-5,7,10], limited data exist from other countries, which likely have different healthcare systems with considerable variation in the quality of care provided by different hospitals. As a part of a quality improvement project, this study examined the outcomes of critically ill patients transferred from other hospitals to a tertiary care ICU in Riyadh, Saudi Arabia.

## Methods

### Design and setting

This was a retrospective cohort study of adult patients who were admitted to the medical-surgical ICU of King Abdulaziz Medical City, Riyadh, Saudi Arabia from March 1999 to December 2010. The ICU is a 21-bed closed unit with a 24 hours/7 days in-house coverage by board-certified intensivists [12] and admits approximately 900 patients per year. The hospital is a tertiary care referral center and is accredited by the Joint Commission International. The study was approved by the Institutional Review Board of the hospital. The consent was waived because of the observational nature of the study.

### Transfer process

Transfer requests are initiated by the referring hospitals or by the patients’ families. A medical report is sent from the referring hospital detailing the medical condition and justification for the transfer. The report is then reviewed by an administrative committee to determine the eligibility of the patient for hospital admission. The concerned services and the ICU team assess eligible patients using the data in the medical report and by phone conversation with the referring physician if required, for potential benefit from the transfer and the stability for the transfer. Once the patient is accepted, the transfer process is initiated. Patients are transferred only if the clinical condition is deemed stable enough as judged by the respiratory, cardiovascular, and other clinical parameters. Transfers occur by fixed-wing airplanes, helicopters, or by ground ambulances depending on the distance from the referring hospitals. The transportation team normally consists of an intensivist or anaesthetist physician and a critical care nurse. The ambulance, helicopter, and fixed-wing airplane are equipped with life support equipments.

### Data collection

We extracted data for this study as a part of a quality improvement project from our ICU database, which recorded all consecutive admissions prospectively by a full-time data collector. The following variables were collected: source of admission to the ICU (other hospitals, emergency department (ED), and hospital wards), age, gender, height, weight, acute physiology and chronic health evaluation (APACHE) II score [13], chronic comorbidities (chronic liver, chronic cardiovascular, chronic respiratory, chronic renal, and chronic immunocompromised) as defined by APACHE II system, admission diagnosis category (respiratory, cardiovascular, neurological, other medical, nonoperative trauma, and postoperative), history of diabetes mellitus, admission postcardiac arrest, and mechanical ventilation (MV). We also documented physiologic and clinical characteristics on the first hour of ICU admission that included heart rate >150 beats/min, hypotension (defined as systolic blood pressure (SBP) <90 mmHg), and acute kidney injury (AKI) (defined as creatinine level >176.8 μmol/L). The following variables were documented during the ICU course: tracheostomy, renal replacement therapy (RRT) using continuous venovenous hemodialysis (CVVHD) or intermittent hemodialysis (HDI), and do-not-resuscitate (DNR) orders.

### Study population

All consecutive patients admitted to the ICU were included in the study. Patients were divided into three groups according to the source of admission to the ICU: transfers from other hospitals, direct admissions from the ED, and admitted from hospitals wards. Patients with direct admission from hospital wards were usually nontrauma patients, because trauma patients come to ICU through ED or from other hospitals. Patients who were admitted from the operating room and recovery room were excluded because of major differences in their course and outcome from transferred patients.

### Outcomes

The primary outcome was the hospital mortality and was available for all patients at the time of hospital discharge and refers to the outcome from King Abdulaziz Medical City. None of the patients were transferred back to the referral center. The secondary outcomes were ICU mortality, ICU and hospital length of stay (LOS), mechanical ventilation duration, and need for tracheostomy and RRT.

### Statistical analysis

Continuous data were presented as means with standard deviations (SD) and categorical data as frequencies and percentages. Chi-square or Student’s *t* test was used to test significant differences between the patients transferred from other hospitals and each of the other two groups as appropriate. To adjust for differences in severity of illness among the groups, standardized mortality ratio (SMR) was calculated by dividing the observed mortality by that predicted by the APACHE II and was reported with its 95% confidence interval (CI) [14]. We also performed stratified analysis for the following admission categories: respiratory, cardiovascular, neurological, other medical, nonoperative trauma, and postoperative. To determine the predictors of hospital mortality among patients transferred from other hospitals, multivariate step-wise logistic regression analyses with hospital mortality as dependent variable were carried out with the following independent variables entered in the model: age, gender, APACHE II score on admission, admission diagnosis category, chronic comorbidities, mechanical ventilation, and admission physiological and clinical variables (coma, heart rate, SBP, and AKI). *P* < 0.05 was considered significant. Statistical analysis software (SAS, version 9.0; SAS Institute, Cary, NC) was used to analyze data.

## Results

### General

During the study period, a total of 7,654 patients were admitted to the ICU, of whom 611 (8%) patients were transferred from other hospitals, 2,703 patients (35.3%) had direct admission from ED and 4,340 patients (56.7%) from hospital wards (Table 1).

**Table 1:** Baseline characteristics of patients transferred from other hospital compared with those with direct admission from emergency department and hospital wards

	**Other hospital**	**ED**	*p *value*	**Hospital wards**	*p *value**
	**(N = 611)**	**(N = 2703)**		**(N = 4340)**	
Age, mean (SD), yr	46.3±22.5	47.9±22.1	0.12	58.2±18.7	<0.0001
Female gender, no. (%)	192 (31.4)	859 (31.8)	0.86	1938 (44.7)	<0.0001
Height (cm), mean (SD)	160.6±24.3	161.0±23.3	0.75	157.8±22.2	0.01
Weight (Kg), mean (SD)	73.6±21.5	72.6±20.7	0.31	69.5±21.1	<0.0001
APACHE II score, mean (SD)	21.7±8.5	21.2±8.8	0.26	26.6±9.0	<0.0001
Chronic co-morbidities, no. (%)					
Chronic liver disease	41 (6.7)	204 (7.7)	0.44	796 (18.5)	<0.0001
Chronic cardiovascular disease	57 (9.4)	388 (14.5)	0.0008	971 (22.6)	<0.0001
Chronic respiratory disease	61 (10)	377 (14.1)	0.008	860 (20)	<0.0001
Chronic renal disease	42 (6.9)	265 (9.9)	0.02	903 (21)	<0.0001
Chronic Immunocompromised	33 (5.4)	158 (5.9)	0.64	662 (15.4)	<0.0001
Admission diagnosis category, no. (%)					
Respiratory	140 (23)	638 (23.6)	0.007	1421 (32.7)	<0.0001
Cardiovascular	141 (23.1)	727 (26.9)		2114 (48.7)	
Neurological	62 (10.2)	304 (11.3)		334 (7.7)	
Other medical	40 (6.6)	185 (6.9)		311 (7.2)	
Non-operative trauma	185 (30.3)	746 (27.6)		29 (0.7)	
Post-operative	42 (6.9)	101 (3.7)		42 (6.9)	
Diabetes mellitus, no. (%)	154 (25.2)	687 (25.4)	0.91	1734 (40)	<0.0001
Admitted post-cardiac arrest, no. (%)	36 (5.9)	172 (6.4)	0.66	439 (10.1)	0.0009
Mechanical ventilation, no. (%)	451 (73.9)	2170 (80.3)	0.0005	2839 (65.5)	<0.0001
Physiologic and clinical characteristics on ICU admission, no. (%)					
Coma (GCS <6)	250 (41.0)	1179 (43.6)	0.23	1430 (33)	<0.0001
Heart rate >150 per minute	18 (3)	103 (3.8)	0.31	324 (7.5)	<0.0001
SBP <90 mm Hg	102 (16.7)	591 (21.9)	0.005	1665 (38.4)	<0.0001
Acute kidney injury, no. (%)	77 (12.6)	380 (14.1)	0.35	1003 (23.1)	<0.0001

*Comparison between patients transferred from other hospitals and ED patients.

**Comparison between patients transferred from other hospitals and hospital wards.

*APACHE* acute physiology and chronic health evaluation, *ICU* intensive care unit, *GCS* Glasgow coma scale, *SBP* systolic blood pressure, *SD* standard deviation.

### Baseline characteristics

Compared with patients who had direct admission from ED, patients transferred from other hospitals were less likely to have cardiovascular (*p* = 0.0008), respiratory (*p* = 0.008), and renal (*p* = 0.02) chronic comorbidities, less likely to be mechanically ventilated (*p* = 0.0005), or to have hypotension on admission (*p* = 0.005; Table 1). Compared with patients with direct admission from hospital wards, patients transferred from other hospitals were younger (*p* < 0.0001), less likely to be females (*p* < 0.0001), had lower APACHE II score (*p* < 0.0001), and less likely to have chronic comorbidities (*p* < 0.0001 for each) or diabetes (*p* < 0.0001) or to be admitted to ICU postcardiac arrest (*p* < 0.0009) and were more likely to be mechanically ventilated (*p* < 0.0001). Additionally, on admission to ICU, they were less likely to have heart rate of >150 beats/minute (*p* < 0.0001), to be hypotensive (*p* < 0.0001), or to have AKI (*p* < 0.0001) compared with patients transferred from hospital wards (Table 1).

### Primary outcome

Hospital mortality was not significantly different in patients transferred from other hospitals compared with those with direct admission from ED (35% vs. 33.1%, *p* = 0.37) but was significantly lower compared with those with direct admission from hospital wards (35% vs. 51.2%, *p* < 0.0001; Table 2). SMRs did not differ significantly across the three groups (0.93, 95% confidence interval [CI] = 0.83-1.03 for patients transferred from other hospitals, 0.91, 95% CI = 0.86-0.96 for patients with direct admission from ED and 0.93, 95% CI = 0.90-0.95 for patients with direct admission from hospital wards; Table 2).

**Table 2:** Outcomes of patients transferred from other hospital compared with those with direct admission from emergency department and hospital wards

	**Other hospital**	**ED**	*p *value*	**Hospital wards**	*p *value**
	**(N = 611)**	**(N = 2703)**		**(N = 4340)**	
Hospital mortality, no. (%)	214 (35.0)	895 (33.1)	0.37	2223 (51.2)	<0.0001
ICU mortality, no. (%)	128 (21.0)	605 (22.4)	0.44	1327 (30.6)	<0.0001
Standardized mortality ratio (SMR) (95% CI)	0.93 (0.83–1.03)	0.91 (0.86–0.96)		0.93 (0.90–0.95)	
ICU LOS (days), mean (SD)	12.1±14.7	8.8±10.5	<0.0001	9.2±52.0	0.17
Hospital LOS (days), mean (SD)	65.5±89.9	39.7±69.8	<0.0001	64.2±101.9	0.76
Mechanical ventilation duration (days), mean (SD)	10.2±13.7	7.9±10.3	<0.0001	7.2±13.6	<0.0001
Tracheostomy, no. (%)	151 (24.7)	539 (19.9)	0.009	629 (14.5)	<0.0001
CVVHD, no. (%)	53 (8.7)	246 (9.1)	0.74	711 (16.4)	<0.0001
Hemodialysis, no. (%)	43 (7.0)	159 (5.9)	0.28	522 (12.0)	0.0003
DNR order, no. (%)	99 (16.2)	553 (20.5)	0.02	1208 (27.8)	<0.0001

*Comparison between patients transferred from other hospitals and ED patients.

**Comparison between patients transferred from other hospitals and hospital wards.

*ICU* intensive care unit, *LOS* length of stay, *CVVHD* continuous venovenous hemodialysis, *DNR* do-not-resuscitate, *SD* standard deviation.

### Secondary outcomes

ICU mortality was not significantly different in patients transferred from other hospitals compared with those with direct admission from ED (21% vs. 22.4%, *p* = 0.44) but was significantly lower compared with those with direct admission from hospital wards (21% vs. 30.6%, *p* < 0.0001). Patients transferred from other hospitals had longer ICU and hospital LOS compared with patients with direct admission from ED (12.1 ± 14.7 days vs. 8.8 ± 10.5 days, *p* < 0.0001 and 65.5 ± 89.9 days vs. 39.7 ± 69.8 days, *p* < 0.0001, respectively) but not different from that of patients with direct admission from hospital wards. Mechanical ventilation duration was longer in patients transferred from other hospitals compared with patients with direct admission from ED (10.2 ± 13.7 days vs. 7.9 ± 10.3 days, *p* < 0.0001) and hospital wards (10.2 ± 13.7 days vs. 7.2 ± 13.6 days, *p* < 0.0001). Moreover, patients transferred from other hospitals required more tracheostomy compared with patients with direct admission from ED (*p* = 0.009) and hospital wards (*p* < 0.0001) and had less DNR orders compared with those with direct admission from ED (*p* = 0.02) and hospital wards (*p* < 0.0001; Table 2).

### Mortality according to admission category

**Figure 1:**
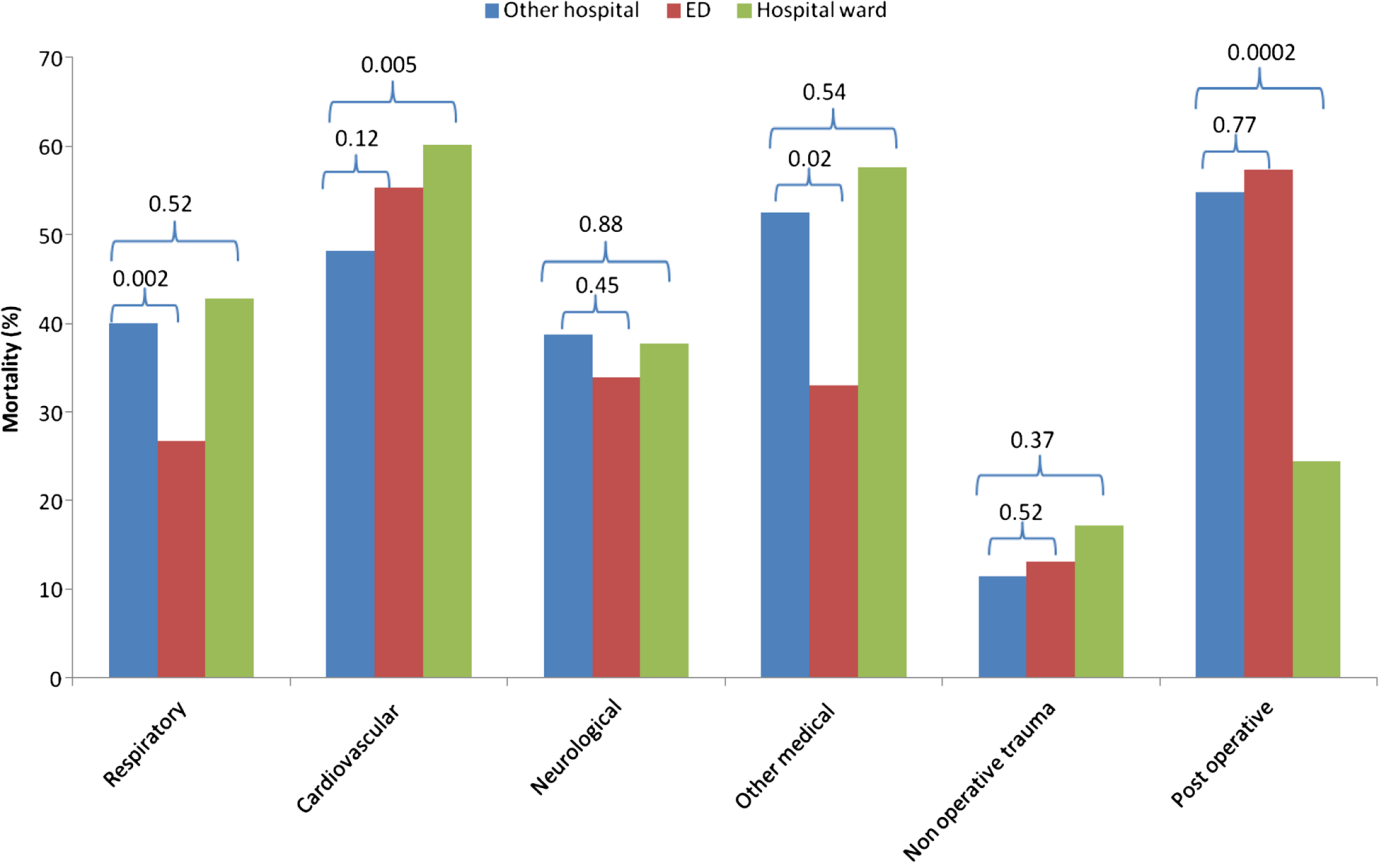
Hospital mortality in patients transferred from other hospitals compared with those with direct admissions from ED and hospital wards according to different admission categories.

Compared with patients with direct admission from ED, patients transferred from other hospitals had higher hospital mortality if they were admitted due to respiratory reason (*p* = 0.002) or due to other medical reason (*p* = 0.02). Compared with patients with direct admission from hospital wards, transferred patients had lower hospital mortality if they were admitted due to cardiovascular reason (*p* = 0.005) but higher if they were admitted postoperatively (*p* = 0.0002; Figure 1).

### Predictors of hospital and ICU mortality

On multivariate analysis, we found the following variables to be independent predictors of hospital mortality among patients transferred from other hospitals: APACHE II score (adjusted odds ratio [aOR] each one-point increment = 1.15, 95% CI = 1.11-1.19, *p* < 0.0001) and chronic liver disease (aOR = 2.99, 95% CI = 1.31-6.8, *p* = 0.009). Young age (aOR = 0.99, 95% CI = 0.97-1.00, *p* = 0.02), APACHE II score (aOR for each one-point increment = 1.2, 95% CI = 1.15-1.25, *p* < 0.0001) and chronic liver disease (aOR = 3.6, 95% CI = 1.56-8.28, *p* = 0.003) were found to be independent predictors of ICU mortality among patients transferred from other hospitals (Table 3).

**Table 3:** Multivariate analyses to determine the predictors of ICU and hospital mortality among patients transferred from other hospitals

	**ICU mortality**		**Hospital mortality**	
	aOR* (95% CI)	***p* value**	aOR* (95% CI)	***p* value**
Age	0.99 (0.97–1.00)	0.02	NS	NS
APACHE II score, each one-point increment	1.2 (1.15–1.25)	<0.0001	1.15 (1.11–1.19)	<0.0001
Chronic liver disease	3.6 (1.56–8.28)	0.003	2.99 (1.31–6.8)	0.009
Female gender	0.56 (0.29–1.05)	0.07	NS	NS

*aOR* adjusted odds ratio, *CI* confidence interval, *NS* not significant, *APACHE* acute physiology and chronic health evaluation.

*The following variables were entered in the model age, gender, APACHE II score on admission, admission diagnosis category, chronic comorbidities, mechanical ventilation, and admission physiological and clinical variables (coma, heart rate > 150 beats/minute, SBP < 90 mm HG, and AKI).

## Discussion

Our study found that patients who were transferred from other hospitals to our center had less hospital and ICU mortality compared with those with direct admission from hospital wards but similar to those with direct admission from ED. Patients transferred from other hospitals had lower severity of illness compared with patients with direct admission from hospital wards but similar to those with direct admission from ED. However, transferred patients did not have higher risk-adjusted mortality compared with nontransferred patients. Increasing severity of illness and chronic liver disease were independent predictors of increased hospital mortality in patients transferred from other hospitals.

There is a considerable variation in transfer practices among centers and countries, which may explain the differences in outcomes observed in different studies. Table 4 summarizes the percentage of transferred patients, transferred population, key transfer process, and main findings in different studies. The mortality rate of transferred patients to our ICU is similar to those admitted from ED but lower than patients with direct admission from the hospital wards. However, risk-adjusted mortality was not different from either group. In contrast, Rosenberg et al. found in a prospective cohort study of 4,579 patients and after comprehensive adjustment that transferred patients had higher ICU and hospital mortality compared with direct admissions [3]. Transferred patients were more likely to have septic shock, ARDS, and comorbid conditions [3]. They also were more likely to be transferred after complications of liver failure [3]. Similarly, Combes et al. retrospectively reviewed 3,416 patients and found that transferred patients had higher ICU and hospital mortality compared with direct admissions. The patients were more likely to have medical admission, higher severity of illness as assessed by SAPS II, and acute respiratory failure [4].

**Table 4:** Comparison of studies from different countries evaluating outcomes of critically ill patients who had interhospital transfer

**Country, author, year**	**Percent of transferred patients of all ICU admissions**	**Transfer population**	**Transfer modality**	**Main findings**
**USA**[3] (Rosenberg et al., Ann Intern Med 2003, **138**(11):882–90)	23%	Medical	Not reported	Risk adjusted mortality was higher in transferred patients compared with direct admissions.
**France**[4] (Combes et al., Crit Care Med 2005, 33(4):705–10)	17%	Medical	Not reported	ICU mortality and SMR was higher for transferred patients compared with direct admissions.
**Canada**[5] (Hill et al., J Crit Care 2007, 22(4):290–5)	20.5%	Medical and trauma	Ground ambulance	Crude ICU and hospital mortality rates were significantly higher in transfer patients compared with patients with direct admission from ED. Adjusted analysis was significant only for ICU mortality but not for hospital mortality.
**USA**[7] (Golestanian et al., Crit Care Med 2007, 35(6):1470–6)	12%	Medical, surgical, and trauma	Not reported	Risk adjusted mortality was similar in transferred and nontransferred patients. Adjusted length of stay was significantly longer only in the transferred group of patients and greater hospital expenditure was associated with transferred patients.
**Canada**[6] (Sampalis et al., J Trauma 1997, 43(2):288–95; 295–6)	37%	Trauma	Air and ground ambulance	Adjusted mortality was higher in patients transferred from other hospitals compared to direct admission to a Level I trauma centre. Adjusted length of ICU and hospital stay was longer in transferred patients compared to direct admissions.
**Saudi Arabia** (our study)	8%	Medical, surgical, and trauma	Air and ground ambulance	Crude hospital and ICU mortality was lower in transferred patients compared with hospital ward patients. However, transferred patients had similar risk-adjusted mortality compared with nontransferred patients.

Our study shows that among all patients admitted to our ICU, only 8% were transferred from other hospitals. This is lower than what has been reported in previous studies where transferred patients constituted up to 23% of all ICU admissions [3,4]. The relatively low rate of transfer to our ICU is likely due to multiple factors. The main reason is probably the limited availability of ICU beds in our hospital. Additionally, our transfer process accepts only patients who are deemed stable enough for transfer. This differs from the practice in United States [15] and Europe [16] where interhospital transfers are performed by hospital-based dedicated teams who transfer patients who might be very unstable. Finally, the limited bed availability results in long waiting time for transfer. As a result, this adds to the selectivity of transfers for patients who survive the initial stage of critical illness.

Trauma was the major admission category among the transferred patients in our cohort. This is a reflection of the fact that trauma is major public health problem in Saudi Arabia and is a leading cause of mortality and morbidity [17,18] and that our hospital is a referral center for trauma. The large number of trauma patients influences the case mix of patients transferred from other hospitals as such patients typically have lower APACHE II scores than patients with other comorbid conditions and lower mortality. The resemblance of transferred patients with ED patients in terms of presentation and severity of illness supports this hypothesis as most of the trauma patients report directly to ED as opposed to patients with other comorbid conditions who will be admitted to hospital wards after initial stabilization.

Our study found that patients transferred from other hospital due to respiratory reason had higher hospital mortality compared with patients with direct admission from ED. This finding is an indication that patients with such medical problems should be considered for transfer to a tertiary care center at an early stage of critical illness and might benefit from advanced health care facilities. Lower hospital mortality due to cardiovascular problems in such patients compared with those with direct admission from hospital wards could simply be due to the fact that only those who survived the initial acute cardiovascular insult were transferred to our hospital while direct admission from hospital wards included all the patients, including postcardiac arrest with expected high mortality.

We also found that patients transferred from other hospitals were more likely to receive tracheostomy. This finding reflects the large number of transferred trauma patients who often require this procedure [19-21]. This also reflects the fact that patients often are transferred after they pass the acute phase of critical illness and are in a stage when tracheostomy is indicated.

Our study should be viewed in terms of its strengths and limitations. In terms of strengths, this is the first study from Saudi Arabia to describe the characteristics and outcomes of patients who had interhospital transfer. The data were collected prospectively by a full-time trained data collector. In terms of limitations, we did not have data regarding the patient condition and severity of illness from the previous hospital. Severity of illness as assessed by APACHE is ideally done for patients within 24 hours of admission to ICU and not on presentation to the referring hospital. However, severity of illness does not accurately reflect the risk of adverse outcomes in transferred patients because of correction of certain physiologic and laboratory abnormalities that artificially improve the physiologic scores before transfer [22]. We do not have data regarding the duration of stay in the other hospitals before the transfer. Also, the selection of patients for transfer as deemed by the clinical condition of the patient might have caused potential selection bias by transferring relatively stable patients while those who were critically ill and not stable enough for transfer and could have been benefitted from advanced health care facilities were not transferred.

## Conclusions

We found that critically ill patients who were transferred to a tertiary care hospital in Saudi Arabia from other hospitals constituted 8% of all ICU admissions and had similar mortality to patients with direct admission from the ED but lower than that of patients with direct admission from the hospital wards. However, risk-adjusted mortality was similar to both groups. Further studies are needed to examine the risks and benefits of transferring less stable patients versus continuing management in their primary hospitals.

## Competing interests

The authors declare that they have no competing interests.

## Authors’ contributions

AHR participated in conception and design, participated in analysis and interpretation of data, drafted the manuscript, critically revised the manuscript for important intellectual content, and approved the final version to be published. ASA participated in analysis and interpretation of data, helped to draft the manuscript, critically revised the manuscript for important intellectual content, and approved the final version to be published. SHH participated in conception and design, helped in drafting the manuscript, critically revised the manuscript for important intellectual content, and approved the final version to be published. HMT helped in statistical analysis and interpretation of data, critically revised the manuscript for important intellectual content, and approved the final version to be published. HMA participated in analysis and interpretation of data, critically revised the manuscript for important intellectual content, and approved the final version to be published. AAJ participated in conception and design, critically revised the manuscript for important intellectual content, and approved the final version to be published. AAS participated in conception and design, critically revised the manuscript for important intellectual content, and approved the final version to be published. MRS participated in acquisition of data, critically revised the manuscript for important intellectual content, and approved the final version to be published. YMA participated in conception and design, acquisition of data, analysis and interpretation of data, critically revised the manuscript for important intellectual content, and approved the final version to be published. All authors read and approved the final manuscript.

## References

[B1] Brogan TV Thiagarajan RR Rycus PT Bartlett RH Bratton SL Extracorporeal membrane oxygenation in adults with severe respiratory failure: a multi-center database Intensive Care Med 2009 3 2105 2114 10.1007/s00134-009-1661-7 19768656

[B2] Peek GJ Mugford M Tiruvoipati R Wilson A Allen E Thalanany MM Hibbert CL Truesdale A Clemens F Cooper N Efficacy and economic assessment of conventional ventilatory support versus extracorporeal membrane oxygenation for severe adult respiratory failure (CESAR): a multicentre randomised controlled trial Lancet 2009 3 1351 1363 10.1016/S0140-6736(09)61069-2 19762075

[B3] Rosenberg AL Hofer TP Strachan C Watts CM Hayward RA Accepting critically ill transfer patients: adverse effect on a referral center’s outcome and benchmark measures Ann Intern Med 2003 3 882 890 10.7326/0003-4819-138-11-200306030-00009 12779298

[B4] Combes A Luyt CE Trouillet JL Chastre J Gibert C Adverse effect on a referral intensive care unit’s performance of accepting patients transferred from another intensive care unit Crit Care Med 2005 3 705 710 10.1097/01.CCM.0000158518.32730.C5 15818092

[B5] Hill AD Vingilis E Martin CM Hartford K Speechley KN Interhospital transfer of critically ill patients: demographic and outcomes comparison with nontransferred intensive care unit patients J Crit Care 2007 3 290 295 10.1016/j.jcrc.2007.06.002 18086399

[B6] Sampalis JS Denis R Frechette P Brown R Fleiszer D Mulder D Direct transport to tertiary trauma centers versus transfer from lower level facilities: impact on mortality and morbidity among patients with major trauma J Trauma 1997 3 288 295 discussion 295–286 10.1097/00005373-199708000-00014 9291375

[B7] Golestanian E Scruggs JE Gangnon RE Mak RP Wood KE Effect of interhospital transfer on resource utilization and outcomes at a tertiary care referral center Crit Care Med 2007 3 1470 1476 10.1097/01.CCM.0000265741.16192.D9 17440423

[B8] Odetola FO Clark SJ Gurney JG Dechert RE Shanley TP Freed GL Effect of interhospital transfer on resource utilization and outcomes at a tertiary pediatric intensive care unit J Crit Care 2009 3 379 386 10.1016/j.jcrc.2008.11.007 19327327

[B9] Marais E de Jong G Ferraz V Maloba B Duse AG Interhospital transfer of pan-resistant acinetobacter strains in Johannesburg, South Africa Am J Infect Control 2004 3 278 281 10.1016/j.ajic.2003.11.004 15292892

[B10] Surgenor SD Corwin HL Clerico T Survival of patients transferred to tertiary intensive care from rural community hospitals Crit Care 2001 3 100 104 10.1186/cc993 11299068 PMC30715

[B11] Hill AD Fowler RA Nathens AB Impact of interhospital transfer on outcomes for trauma patients: a systematic review J Trauma 2011 3 1885 1900 discussion 1901 10.1097/TA.0b013e31823ac642 22182900

[B12] Arabi Y Pro/Con debate: should 24/7 in-house intensivist coverage be implemented? Crit Care 2008 3 216 10.1186/cc6905 18557996 PMC2481458

[B13] Knaus WA Draper EA Wagner DP Zimmerman JE APACHE II: a severity of disease classification system Crit Care Med 1985 3 818 829 10.1097/00003246-198510000-00009 3928249

[B14] Goldhill DR Sumner A Outcome of intensive care patients in a group of British intensive care units Crit Care Med 1998 3 1337 1345 10.1097/00003246-199808000-00017 9710091

[B15] Warren J Fromm RE Jr Orr RA Rotello LC Horst HM Guidelines for the inter- and intrahospital transport of critically ill patients Crit Care Med 2004 3 256 262 10.1097/01.CCM.0000104917.39204.0A 14707589

[B16] Intensive Care Society Guidelines for the transport of the critically ill adult http://criticalcaremedicine.pbworks.com/f/Transport+of+Critically+Ill+Patient~ICS.PDF. Accessed on July 20, 2012

[B17] Shanks NJ Ansari M al-Kalai D Road traffic accidents in Saudi Arabia Public Health 1994 3 27 34 10.1016/S0033-3506(05)80032-0 8202582

[B18] Ansari S Akhdar F Mandoorah M Moutaery K Causes and effects of road traffic accidents in Saudi Arabia Public Health 2000 3 37 39 10787024 10.1038/sj.ph.1900610

[B19] Branco BC Plurad D Green DJ Inaba K Lam L Cestero R Bukur M Demetriades D Incidence and clinical predictors for tracheostomy after cervical spinal cord injury: a national trauma databank review J Trauma 2011 3 111 115 10.1097/TA.0b013e3181d9a559 20526209

[B20] Shirawi N Arabi Y Bench-to-bedside review: early tracheostomy in critically ill trauma patients Crit Care 2006 3 201 10.1186/cc4548 16356202 PMC1550867

[B21] Arabi Y Haddad S Shirawi N Al Shimemeri A Early tracheostomy in intensive care trauma patients improves resource utilization: a cohort study and literature review Crit Care 2004 3 R347 R352 10.1186/cc2924 15469579 PMC1065024

[B22] Durairaj L Will JG Torner JC Doebbeling BN Prognostic factors for mortality following interhospital transfers to the medical intensive care unit of a tertiary referral center Crit Care Med 2003 3 1981 1986 10.1097/01.CCM.0000069730.02769.16 12847392

